# Water-related atmospheric agents and solubility: two parameters of validation in toxicological screening on clothing worn by skeletal remains

**DOI:** 10.1007/s00414-023-02997-0

**Published:** 2023-04-13

**Authors:** Gaia Giordano, Giulia Caccia, Michele Boracchi, Francesco Sardanelli, Cristina Cattaneo, Domenico Di Candia

**Affiliations:** 1 Dipartimento Di Scienze Biomediche per La Salute, Università Degli Studi Di Milano, Via Luigi Mangiagalli 37, 20133 Milan, Italy; 2 Dipartimento di Medicina Sperimentale, Università degli Studi della Campania Luigi Vanvitelli, Napoli, Italy

**Keywords:** Rainfall simulation, Rainfall intensities, Molecules solubility, Fabrics remains as toxicological matrix, Toxicological analyses on skeletal remains

## Abstract

In forensic toxicology, when conventional matrices are no longer available, alternative matrices can be used to assess toxicological investigations. Clothes worn by skeletal remains may be a good unconventional matrix for toxicological analyses considering that they have absorbed decomposition fluids and blood from a body. We hypothesized a scenario in which a skeleton, wearing clothes, was discovered in an open environment. From this starting point, an experimental study was developed on different textiles (cotton, wool, and polyester) to evaluate whether water-related atmospheric agents and molecule solubility can largely influence the detection of molecules of toxicological interest on this specific matrix, together with the characteristics of different garments chosen. The experimental study was performed on blood spots, previously spiked with 6-monoacetylmorphine and morphine, accurately placed on different textiles and washed with different quantities of deionized water adjusted at pH 5.6 with formic acid to simulate different rainfall conditions. Toxicological analyses were performed via Solid-Phase Extraction and High-Performance Liquid Chromatography—Tandem Mass Spectrometry analyses (Thermo Scientific™ TSQ Fortis™ II Triple-Quadrupole Mass Spectrometer). From the experimental study morphine could not be detected on 100% cotton and 100% wool fabric after the passing of 500 mL of deionized water and in 100% synthetic polyester textile after washing with 250 mL of deionized water. In conclusion, when toxicological analyses are carried out on unconventional matrices as textiles worn by corpses exposed to different environmental conditions, it is of great importance, in using such substrates as evidence for the presence of molecules of toxicological interest, to evaluate chemical-physical characteristics of each analyte under investigation in order to correctly interpret the toxicological data obtained.

## Introduction

Forensic toxicological analyses are often involved in forensic sciences with the aim to investigate the presence of xenobiotics on conventional matrices as biological fluids or samples of viscera [1–3]. When conventional matrices are no longer available for analytical investigation, often due to complete skeletonization, alternative matrices can be used in forensic toxicology to replace conventional ones. Thus, when the conventional matrices are no longer available, unconventional matrices such as bones, nails, and teeth can be involved in toxicological analyses [4, 5] to investigate chronic intake of drugs. Still considering the same scenario in which an extremely decomposed or even complete skeletonized body is discovered, the presence of alternative matrices that could account for the acute administration of substances is extremely rare.

For this reason, if some clothes that are worn by a skeleton are discovered in a forensic scenario, these can be collected and analyzed with the aim to assess a possible detection of substances of toxicological interest near the time of death of an individual, considering that the clothes have been soaked into decomposed blood and/or fluids of the body in decomposition.

We performed an in-depth literature review on the main databases, PubMed and Science Direct, performing advanced research by Title/Abstract and including papers published since the year 2000. During the literature review, different query strings were applied for the research, such as “toxicology clothing”, “toxicology fabric” and “toxicology textile”. The results obtained for the query string “toxicology clothing” reported 73 results in PubMed and 32 in Science Direct. Most of the obtained results were excluded considering that they did not deal with toxicological analyses on textiles. However, four of them were considered appropriate due to the fact that they discussed toxicological analyses performed on clothing matrices. One study, even if not related to our aim, considered the exposure of individuals to heavy metals that can be detected in textile material [6], another study investigated how dyes of cotton fibers can migrate from clothes and penetrate into human skin due to sweat composition and sweat pH in situations of intense perspiration on the living [7]. Whereas, authors of other two researches have extracted azo dyes from different clothes types [8, 9]. The query string “toxicology fabric” resulted in 45 papers in PubMed and 28 results in Science Direct. From these, another paper was taken into consideration during our literature review because it investigated the presence of trace elements and indigo dye in denim garments [10]. From the query string “toxicology textile” no papers discussing toxicological analyses performed on clothes were noticed. Therefore, in literature, no studies of research of drugs on different clothes or textiles seemed to have been performed; experimental studies have been carried out on this type of substrate concerning only the presence of DNA traces on cotton cloths [11–13] and on polyester textiles [13], obtaining positive findings.

Therefore, we developed an experimental study to evaluate if fabric remains in hypothesis worn by a skeletonized individual, found in an open environment after a very long post-mortem interval, can be a good and innovative unconventional matrix for toxicological analyses, even after exogenous stress such as water-related atmospheric interferences. This would never replicate what happens during exposure, however it would give an idea of whether substances of toxicological interest planted on fabric in theory can resist drastic immersion in water.

Toxicological analyses were performed with Solid-Phase Extraction technique reported in the material and methods section together with validation procedures. The instrumental conditions of High-Performance Liquid Chromatography-Tandem Mass Spectrometry (HPLC–MS/MS) system TSQ Fortis II are detailed in our previous papers [14, 15].

### Hypothesis

We developed an experimental study to investigate the detectability of two molecules (6-monoacetylmorphine (6-MAM) and morphine) on clothing undergoing the effects of water-related atmospheric agents. Apart from the influence of post-mortem interval, water-related atmospheric agents as rain and humidity could influence the determination of these two molecules differently. We selected these substances due to the fact that they are commonly detected in autopsy cases in our region of interest [15] and considering that morphine is 9.03 times more soluble than 6-MAM. In fact, water solubility of 6-MAM is 1.14 mg/mL, whereas the solubility of morphine is 10.3 mg/mL. Therefore, we wondered if this difference in solubility could affect the detection of 6-MAM and morphine in three different types of textiles.

## Experimental protocol

### Study plan

**Fig. 1:**
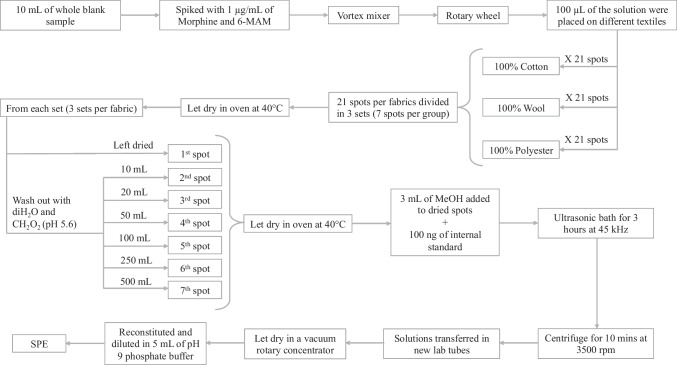
Schematic representation of study plan

We decided to produce several spots of blank blood samples spiked with standard solutions of 6-MAM and morphine on different textiles. The textiles selected were 100% cotton, 100% wool and 100% synthetic polyester fabrics to evaluate the difference between the mechanic absorption of blood due to different types of textiles. Spots of spiked whole blood were left to dry in an oven at 40 °C, washed out with different quantities of slightly acidic deionized water (from dry to 500 mL) to simulate different rainfall intensities extensively explained below (Fig. 1). Deionized water (diH_2_O) was adjusted at pH 5.6 with formic acid in order to reproduce rainwater conditions [16].

## Material and methods

### Instruments involved

A standard 12-port vacuum manifold and Bond Elut™ Certify cartridges 130 mg (Agilent) were used for SPE (Solid Phase Extraction) extraction procedures. Samples were analyzed with a Thermo Scientific™ TSQ Fortis™ II Triple-Quadrupole Mass Spectrometer.

### Chemicals and Reagents

All the standards molecules (morphine 1 mg/mL in methanol and 6-MAM 1 mg/mL in acetonitrile) involved in this study were purchased from Sigma-Aldrich and stored at -20 °C. The Internal Standard (IS) SKF 525-A (Proadifen hydrochloride, analytical standard, > 95%, 100 mg) was purchased from Sigma-Aldrich as well.

Working solutions of each molecule and IS were prepared with methanol or acetonitrile at 100 µg/mL and 1 µg/mL, starting from standard solutions, and stored at -20 °C until use.

Solvents used in the extraction processes were purchased from Sigma-Aldrich (methanol) and from PanReac AppliChem ITW Reagents (phosphate buffer solution pH 4, phosphate buffer solution pH 9 and water for UV, HPLC, ACS).

### Sample collection

A sample of blank femoral blood was collected during autopsy examination. The whole blood specimen was sampled with a sterilized syringe, stabilized with sodium fluoride and potassium oxalate, and analyzed to evaluate the absence of any molecule of toxicological interest.

Textiles selected for this study were 1 square meter (sqm) of 100% cotton UNI EN ISO 13688, 100% wool UNI EN ISO 13934, 100% synthetic polyester UNI EN ISO 12127 respectively (Fig. 1).

### Sample preparation

**Fig. 2:**
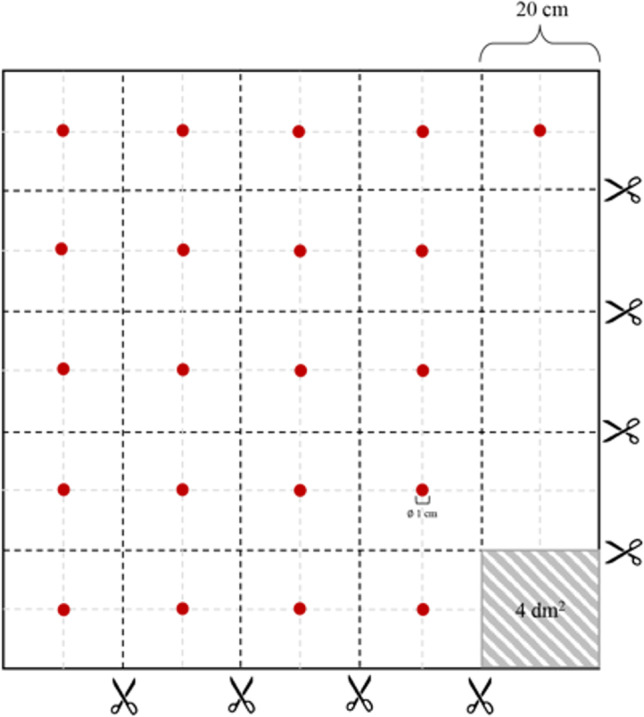
Representation of blood spots located on 1 square meter of textile

Ten milliliters of blank whole blood were placed in a 15 mL Pyrex glass tube and spiked with a final concentration of 1 µg/mL of morphine and 6-MAM, agitated on a Vortex mixer (Heidolph, REAX top), and then placed on a rotating wheel (Falc F205) for 1 h. Three different textiles (cotton, wool, and synthetic polyester fabric) were spotted 21 times (per cloth) with 100 µL of this final solution (each spot contained an effective quantity of 100 ng of 6-MAM and 100 ng of morphine), taking care not to let blood penetrate the fabric in order to avoid loss of matrix. A total of 63 spots were obtained, each of about 1 cm of diameter on all fabrics. Blood-spotted textiles were let dry in an oven at 40 °C until complete dryness (Fig. 1). Each blood spot was located in the middle of a four-square decimeter and separated from other spots by another four-square decimeter. Thus, in a square meter of fabrics, the spots were divided into 4 lines (5 spots per line) plus 1 line with a single spot, obtaining a total of 21 spots per type of textile, as represented in Fig. 2. Each spot was then separated from the other spots by cutting the fabric, leaving the spot in the middle of the four-square decimeter, to avoid a possible contamination from one spot to another.

From each piece of fabric, the spots were divided in 3 sets (7 spots per group). In each set the spots were treated differently: 1 spot remained unwashed and the other 6 were secured on a beaker with a margin of blank fabric, with the spot in the middle of the beaker’s opening.

The six spots were washed out with different quantities of deionized water adjusted at pH 5.6 with formic acid (10 mL, 20 mL, 50 mL, 100 mL, 250 mL, 500 mL). The wash out was performed with a continuous gentle stream of water directed on the blood spot. Subsequently, the sets were let dry in an oven at 40 °C until complete dryness. This process was repeated for all the sets of each textile (Fig. 1).

After drying, we cut the exceeding blank fabric of each sample, paying attention not to cut the margin of the spots. Samples of fabrics were transferred in a 15 mL Pyrex glass tube; 3 mL of methanol were added to the fabrics together with 100 ng of IS. The Pyrex glass tubes were left in ultrasonic bath (USC 600 T, VWR Avantor) for 3 h at 45 kHz (Fig. 1).

### Sample preparation for SPE technique

After ultrasonic bathing, all the samples were centrifuged for 10 min at 3500 rpm (Thermo Scientific, Heraeus Biofuge primo centrifuge), and the 3 mL of methanol was transferred with Pasteur glass pipettes from the Pyrex glass tubes to PP lab tubes, let dry in a vacuum rotary concentrator (Thermo Scientific, Savant SpeedVac Concentrator) and then prepared for SPE extraction with a dilution in 5 mL of pH 9 phosphate buffer, agitated on a Vortex mixer and placed on the rotating wheel for 1 h (Fig. 1).

### SPE extraction

The solutions obtained were loaded on Bond Elut™ Certify cartridges 130 mg (Agilent) previously conditioned with 2 mL of methanol and 2 mL of pH 9 phosphate buffer. After the wash out with 2 mL of water, 2 mL of pH 4 and 2 mL of methanol, the cartridges were left to dry for 30 min at reduced pressure. As a last step, the cartridges were eluted with 2 mL of methanol at 2% ammonium hydroxide (98:2, v/v).

The eluates obtained were let dry in the vacuum rotary concentrator, restored with 100 µL of methanol and 2 µL of these final solutions were analyzed in triplicates via a Thermo Scientific™ TSQ Fortis™ II Triple-Quadrupole Mass Spectrometer. The instrumental conditions of HPLC–MS/MS are reported in our previous paper [14, 15].

### Validation procedures

Validation procedures were performed according to guidelines [17]. Bias and precision were calculated thanks to blank whole blood samples spiked with 6-MAM and morphine using 3 separate samples at low, medium, and high concentrations (3–80-160 ng/mL) over five different runs. The bias was lower than ± 20% at each concentration, whereas within-run precision and between-run precision were calculated by ANOVA Single Factor Calculations approach for all the molecules investigated in this study with a %CV <  ± 20%. A linear calibration model was prepared for 6-MAM and morphine with 7 non-zero calibration points replicated for 5 runs. Calibration curves were developed starting from working solutions with the same ranges: 1–5-10–20-50–100-200 ng/mL and the coefficient of determination (r^2^) for linear calibration model was calculated ≥ 0.99 for each molecule. One-hundred microliters of blank whole blood were fortified with proper amount of morphine and 6-MAM and with 100 ng of IS to obtain 7 non-zero calibration points. The samples were treated as explained previously in this paper, precisely in paragraph “*Sample preparation for SPE technique*” and “*SPE extraction*”.

The carryover effect was investigated by injecting in triplicates an extracted blank sample of whole blood after each highest calibration point. It was noted that carryover was not present for 6-MAM, morphine, or the IS in any of the extracted blank samples. Ten different sources of blank whole blood were analyzed to evaluate matrix interferences; the matrices were extracted without the addition of IS and analyzed. No interferences at the retention time of the molecules under investigation were observed after the analyses of the blank whole blood. Moreover, one matrix was randomly selected, fortified with the IS, extracted, and analyzed to demonstrate the absence of interferences at the retention time of the molecules under investigation by the IS. Another blank matrix sample was spiked with 6-MAM and morphine at the highest calibration point and analyzed without the IS to evaluate if unlabeled analyte ions interfere with the signal of the IS and if the two analytes could interfere to each other. At the end, no interferences were observed. The post-column extraction approach was chosen to perform the ionization suppression/enhancement procedure and two sets of samples were involved for this experiment. The first set consisted in standards prepared in mobile phase at low and high concentration and with 100 ng of IS, not extracted, but simply injected six times each.

The second set consisted in ten different blank samples of whole blood (the same used for interference studies) extracted in duplicate and then spiked with low and high concentration of the analytes and with 100 ng of the IS. Each concentration set sample was injected once. At the end, ionization suppression/enhancement do not exceed the 25% and the %CV was ≤ 20. The LOD was determined as the lowest concentration with a signal-to-noise (S/N) ratio of the peak areas ≥ 3 (0.3 ng/mL) and with all the acceptance criteria met (retention time, peak shape, mass spectral ion ratio), after the analysis of three sources of blank whole blood matrix fortified at decreasing concentrations and analyzed in duplicates over three runs.

The LLOQ was determined as the lowest non-zero calibration point. Three different matrix sources were fortified with the analytes at 1 ng/mL and were analyzed over three runs demonstrating that all criteria were met (detection, identification, bias and precision) and S/N ratio of the peak areas ≥ 10 (1 ng/mL).

## Results

Each spot was analyzed, and the results are summarized in Tables 1, 2 and 3. Tables report the results obtained after the application of experimental conditions previously described. The results obtained from cotton fabric are listed in Table 1; the data recorded from wool are reported in Table 2 and the results noted from synthetic textile are listed in Table 3. The data obtained highlighted the presence of 6-MAM until the 7^th^ spot (with a wash out at 500 mL of diH_2_O adjusted at pH 5.6 with formic acid) in all the set analyzed of all fabrics (quantifiable in cotton and wool, and in non-quantifiable traces in synthetics) (Tables 1, 2 and 3), morphine could not be detected in the last spot of cotton and wool textile (Tables 1 and 2) and could not be detected in the 6^th^ and 7^th^ spot positioned on the synthetic polyester fabric (Table 3).

**Table 1:** Quantitative results of different set and the mean concentration obtained between three sets on cotton fabric

*COTTON*	*Set 1 (ng)*		*Set 2 (ng)*		*Set 3 (ng)*		*Mean (ng)*	
*Spots*	6-MAM	Morphine	6-MAM	Morphine	6-MAM	Morphine	6-MAM	Morphine
*1*^*st*^* (dried)*	96.8	94.1	97.3	94.8	96.2	96.5	**96.8**	**95.1**
*2*^*nd*^* (10 mL)*	89.4	74.2	89.0	73.9	88.5	75.3	**89.0**	**74.5**
*3*^*rd*^* (20 mL)*	85.1	53.4	84.8	51.7	83.2	54.2	**84.4**	**53.1**
*4*^*th*^* (50 mL)*	72.9	49.1	75.8	47.2	73.0	48.3	**73.9**	**48.2**
*5*^*th*^* (100 mL)*	65.9	20.4	69.1	19.6	66.4	18.8	**67.1**	**19.6**
*6*^*th*^* (250 mL)*	44.6	Traces	41.4	Traces	40.8	Traces	**42.3**	**Traces**
*7*^*th*^* (500 mL)*	26.7	ND	25.3	ND	25.9	ND	**26.0**	**ND**

**Table 2:** Quantitative results of different set and the mean concentration obtained between three sets on wool textile

*WOOL*	*Set 1 (ng)*		*Set 2 (ng)*		*Set 3 (ng)*		*Mean (ng)*	
*Spots*	6-MAM	Morphine	6-MAM	Morphine	6-MAM	Morphine	6-MAM	Morphine
*1*^*st*^* (dried)*	97.2	96.1	97.9	96.5	96.3	97.1	**97.1**	**96.6**
*2*^*nd*^* (10 mL)*	90.2	82.0	90.6	81.7	91.0	82.3	**90.6**	**82.0**
*3*^*rd*^* (20 mL)*	85.9	65.2	83.2	64.8	84.9	65.4	**84.7**	**65.1**
*4*^*th*^* (50 mL)*	70.2	43.0	69.3	42.1	69.9	41.9	**69.8**	**42.3**
*5*^*th*^* (100 mL)*	52.0	22.3	51.4	23.0	52.9	22.4	**52.1**	**22.6**
*6*^*th*^* (250 mL)*	46.0	Traces	46.8	Traces	45.4	Traces	**46.1**	**Traces**
*7*^*th*^* (500 mL)*	30.7	ND	28.9	ND	27.4	ND	**29.0**	**ND**

**Table 3:** Quantitative results of different set and the mean concentration obtained between three sets on synthetic polyester fabric

*SYNTHETICS*	*Set 1 (ng)*		*Set 2 (ng)*		*Set 3 (ng)*		*Mean (ng)*	
*Spots*	6-MAM	Morphine	6-MAM	Morphine	6-MAM	Morphine	6-MAM	Morphine
*1*^*st*^* (dried)*	98.2	97.0	97.9	96.3	98.1	96.0	**98.1**	**96.4**
*2*^*nd*^* (10 mL)*	81.2	64.0	81.0	63.9	78.9	64.3	**80.4**	**64.1**
*3*^*rd*^* (20 mL)*	64.3	30.9	64.8	29.6	64.0	29.4	**64.4**	**30.0**
*4*^*th*^* (50 mL)*	53.2	9.8	54.0	10.0	52.9	9.4	**53.4**	**9.7**
*5*^*th*^* (100 mL)*	34.2	Traces	34.8	Traces	33.7	Traces	**34.2**	**Traces**
*6*^*th*^* (250 mL)*	21.0	ND	20.7	ND	19.8	ND	**20.5**	**ND**
*7*^*th*^* (500 mL)*	Traces	ND	Traces	ND	Traces	ND	**Traces**	**ND**

## Discussion

An experimental study was developed to evaluate if fabrics worn by a skeletonized individual, found in an open environment after a very long post-mortem interval could represent a new unconventional matrix for toxicological analyses, even after the interferences due to atmospheric agents such as intensive rain and humidity. The study plan was developed using three different types of textiles (100% cotton, 100% wool and 100% synthetic polyester) spotted with blank whole blood spiked with 6-MAM and morphine. Spiked blood spots were therefore treated differently: one spot (from each fabric) was let dry and subsequently analyzed, whereas the other spots were washed out with different quantities of slightly acidic deionized water (adjusted to pH 5.6 with formic acid to reproduce rainwater conditions) to simulate different rainfall amounts. Our initial assumption, in which the difference of solubilities could lead to quicker loss of morphine if compared to the loss of 6-MAM, could be confirmed by the experimental results. It could be likely that a greater amount of water would be necessary to wash out all the 6-MAM trapped inside the fabric remains with respect to the volumes of water applied to wash out the morphine. The dissimilarities in detection and quantitation of these two molecules in different types of clothes could be explained, in our opinion, by the intrinsic fabrics’ characteristics. Indeed, the major loss of 6-MAM and morphine in polyester fabric could be explained by the hydrophobic characteristic of this textile. Indeed, the synthetic textile can absorb the water only on its surface, resulting in a minor absorption of it and in an easier wash out of the analytes. On the contrary, wool and cotton have given superimposable results holding the molecules with the same strength, especially 6-MAM. These results can be explained thanks to the peculiar characteristics of natural fibers being hydrophilic, hygroscopic and water-absorbing, allowing rainwater to be absorbed within the structure of the fiber and retaining the molecules inside the fabric [18].

## Conclusions

In this research, we developed an experimental study performing toxicological analyses on different types of fabrics (100% cotton, 100% wool and 100% synthetic polyester) to evaluate the different solubility and different stability of molecules on different types of fabric. The results obtained supported our hypothesis and we noticed that intrinsic fabric differences could influence the presence of molecules on textiles: for example, morphine was lost very quickly on synthetic clothing (at 250 mL of diH_2_O at pH 5.6).

In conclusion, this study has shown that clothes belonging to skeletonized individuals may be used in cases in which the conventional and more standardized biological matrices are no longer available for toxicological analyses obtaining important information about the molecules present in the individuals near the time of death (acute administration xenobiotics) and therefore in circulation and present in the putrefactive fluids of soft tissues permeating the clothes. Moreover, we would suggest, considering our toxicological results, that the chemical-physical characteristics of each analyte under investigation as well as the fabric composition that can influence the detection of some analytes should be considered when toxicological analyses are performed on such unconventional matrices.

## Data Availability

The data underlying this article are available in the article and from the corresponding author on reasonable request.
